# Discovery of *Japalura chapaensis* Bourret, 1937 (Reptilia: Squamata: Agamidae) from Southeast Yunnan Province, China

**DOI:** 10.24272/j.issn.2095-8137.2017.064

**Published:** 2018-03-07

**Authors:** Kai Wang, Ke Jiang, Yu-Fan Wang, Nikolay A. Poyarkov, Jing Che, Cameron D. Siler

**Affiliations:** 1 Sam Noble Oklahoma Museum of Natural History & Department of Biology, University of Oklahoma, Norman Oklahoma 73072-7029, USA; 2 Kunming Institute of Zoology, Chinese Academy of Sciences, Kunming Yunnan 650223, China; 3 Southeast Asia Biodiversity Research Institute, Chinese Academy of Sciences, Yezin Nay Pyi Taw 05282, Myanmar; 4 Zhejiang Forest Resource Monitoring Center, Hangzhou Zhejiang 310020, China; 5 Department of Vertebrate Zoology, Biological Faculty, Lomonosov Moscow State University, Leninskiye Gory, GSP-1, Moscow 119234, Russia; 6 Joint Russian-Vietnamese Tropical Research and Technological Center, 63 Nguyen Van Huyen Road, Nghia Do, Cau Giay, Hanoi, Vietnam

**Keywords:** Draconinae, Indochina, Morphological Variation, New record, Range extension, Systematics, Taxonomy

## Abstract

Due to a paucity of surveys in northern Indochina and lack of international collaborations among neighboring countries, recognized distributional ranges for many amphibian and reptile species end at the political borders for some countries, despite seemingly continuous suitable habitat spanning the region. Combining both morphological and genetic data, we report the first discovery of *Japalura chapaensis*, a rare agamid lizard believed previously to be endemic to northern Vietnam only, along the border region of southeastern Yunnan Province, China. To facilitate future research on the genus *Japalura sensu lato* in Indochina, we provide detailed descriptions of additional specimens of this rare species, including the first description of coloration in life and an expanded diagnosis, and discuss the species boundary of *J. chapaensis* with respect to its congeners.

## INTRODUCTION

Species with distributions spanning the political jurisdictions of multiple countries pose special challenges to biodiversity and conservation studies. Not only are conservation assessments and protection of this class of evolutionary lineages more difficult, but also, variation in the understanding of regional biodiversity can lead to conflicting or questionable taxonomic and biogeographic patterns for widespread species. This situation has been particularly pronounced in peripheral island systems, including the Philippines (Esselstyn et al., 2004; Esselstyn & Oliveros, 2010; Siler et al., 2014), eastern Indonesia (Taylor, 2003), and Hawaii (Gillespie et al., 2012), among others (Gillespie & Claque, 2009). However, even for species with broad geographic distributions on continental landmasses, the paucity of communication across political boundaries has resulted in species being recognized as restricted to one side of a country’s border or the other, despite evidence of continuous distributions spanning the boundaries of multiple countries (Dudgeon et al., 2006; Hannah et al., 2002). This is exemplified in regions of Southeast Asia, particularly northern Indochina.

The border between southern China and northern Vietnam has posed problems for studies of amphibian and reptile diversity. Historically, biodiversity research on the herpetofauna across this region has been conducted independently in China and Vietnam, with interpretation of research often slowed by language barriers and lack of regional collaboration (Ananjeva et al., 2007; Bain et al., 2009; Yang & Rao, 2008; Zhao et al., 1999). However, despite the similar habitat structures across this region, and evidence of continuous distributions for ecologically similar species of frogs and lizards (Bain et al., 2009; Ota, 2000), much of the herpetofauna is still thought to be endemic to one side of the political border or the other. An example of this is the Chapa Mountain Dragon, *Japalura chapaensis* Bourret, 1937.

First described as a subspecies of *Japalura swinhonis* Günther, 1864, the description of *J. chapaensis* was based originally on a single female specimen (MNHN 1948.45) from Chapa (now “Sa Pa”) in Lao Cai Province, northern Vietnam, close to the China-Vietnam border (Bourret, 1937). Ota (1989) later elevated *J. chapaensis* to full species based on examination of morphological characters, and three years later, Ota & Weidenhöfer (1992) described the first and only male specimen of the species (KUZ 20097) from the type locality. However, despite some descriptive comparisons to morphologically similar species, low sample size (two specimens of *J. chapaensis*, both with incomplete tails reported) and limited diagnostic analysis against other congeners in the region, has limited our understanding of the phenotypic variation and diagnostic features of *J. chapaensis* (Ota, 1989; Ota & Weidenhöfer, 1992). Currently, *Japalura chapaensis* is known from only two voucher specimens only, with distribution records limited to the eastern slopes of the Hoang Lien Son mountain ridge in northern Vietnam (Ananjeva et al., 2007; Bourret, 1937; Cai et al., 2015; Manthey, 2010; Van Sang et al., 2009). However, as the Hoang Lien Son Mountains continue and extend to the southern part of the Yunnan Province in China, thus offering similar habitats in close geographic proximity to the type locality of *J. chapaensis* in northern Vietnam, it remains possible that *J. chapaensis* also occurs across the border in the southern edge of China. 

**Figure 1:**
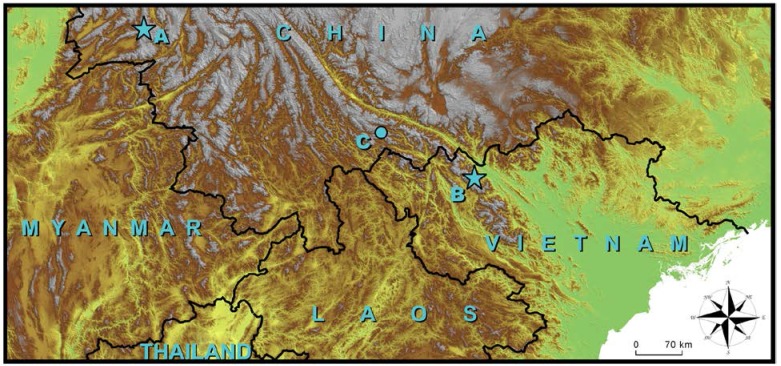
Distribution of *Japalura sensu lato* in northern Indochina

In November 2015, we collected three agamid lizards from Lvchun, Honghe Prefecture, Southern Yunnan, China, close to the China-Vietnam border (Figure 1). Based on newly collected genetic and morphological datasets, we confirmed the identity of these Chinese specimens as *J. chapaensis*. Here, we extend the recognized distribution of *J. chapaensis* into China, provide a description of additional specimens of this rare species, and discuss morphological variation of *J. chapaensis* with relation to mainland congeners, particularly *J. yunnanensis*.

Stars indicate type localities for *J. yunnanensis* (A: Longling, Baoshan, Yunnan Province, China) and *J. chapaensis* (B: Sa Pa, Lào Cai Province, Vietnam); circle shows new locality of *J. chapaensis* (C: Lvchun, southern Yunnan, China).

## MATERIALS AND METHODS

### Field sampling

A total of three specimens of *J. chapaensis* (an adult male, an adult female, and a sub-adult female) were collected from Lvchun, Honghe Prefecture, southern Yunnan Province, China on 10 November 2015 (N22.994 1°, E102.398 9°, 1 550 m elevation, WGS 84), and a topotypic newborn was collected from the vicinity of Tram Don mountain pass, Hoang Lien Son National Park, Sa Pa, Lao Cai Province, Vietnam (GPS N22.353 1°, E103.774 9°, 1 950 m elevation, WGS 84; Figure 1). After euthanasia, tissue samples were taken from the liver and preserved in 95% ethanol, and the voucher specimens were fixed in 10% buffered formalin and transferred to 70% ethanol after fieldwork. All Chinese specimens were deposited at the Museum of Kunming Institute of Zoology, Chinese Academy of Sciences, and the Vietnamese specimen was deposited at the Zoological Museum of Lomonosov Moscow State University, Moscow, Russia.

### Morphometrics

We examined all morphological characters used in previous descriptions of *J. chapaensis* (Bourret, 1937; Ota, 1989; Ota & Weidenhöfer, 1992), as well as additional characters following Wang et al. (2016). Morphometric data were recorded by JK using a digital caliper to the nearest 0.01 mm, except for tail length, which was recorded using a ruler to the nearest 1 mm. The following morphological characters were examined: snout–vent length (SVL), tail length (TAL), trunk length (TRL), head width (HW), snout–eye length (SEL), interorbital distance (IOD), fore-limb length (FLL), hind limb length (HLL), Toe IV length (T4L), Finger IV subdigital lamellae count (F4S), supralabial count (SL), infralabial count (IL), middorsal scale count (MD), Toe IV subdigital lamellae count (T4S), number of scales between nasal and first supralabial (NSL), supraciliary count (SCL), number of scale rows between the sixth supralabial and orbit circle (SOR), enlarged, conical, occipital scale count (COS), enlarged, conical, post-tympanic scale count (PTY), and enlarged, conical, postrictal scale count (PRS).

Summaries of specimens examined are presented in Appendix. For comparisons with other phenotypically similar species, morphological data were collected from type or topotype specimens when available. In addition to the vouchered specimens examined, morphological data of congeners were obtained from literature (Manthey et al., 2012; Ota & Weidenhöfer, 1992). For maximum comparability and consistency, color description terminology followed Köhler (2012). Museum abbreviations followed Sabaj (2016), including: Chengdu Institute of Biology, Chinese Academy of Sciences (CIB); Kunming Institute of Zoology, Chinese Academy of Sciences, Kunming, China (KIZ); Department of Zoology, Kyoto University, Kyoto, Japan (KUZ); National Museum of Natural History, Washington D.C., USA (NMNH); Museum of Comparative Zoology, Harvard University, Cambridge, USA (MCZ); California Academy of Sciences, San Francisco, USA (CAS); National Museum of France, Paris, France (MHNP); and Zoological Museum of Lomonosov Moscow State University, Moscow, Russia (ZMMU).

### DNA sequencing and genetic divergences

We extracted total genomic DNA from the newly collected tissues of *J. chapaensis* from China (KIZ 034923 and KIZ 034921), topotypic *J. chapaensis* (ZMMU NAP-01911), and topotypic *J. yunnanensis* (CAS 242271), using a guanidine thiocyanate extraction protocol. A fragment of 432 bp of the mitochondrial NADH dehydrogenase subunit 2 (*ND*2) gene was targeted and amplified using the primers and protocols of Macey et al. (2000). Previously published sequence data *ND*2 for congeners *J. splendida* and *J. flaviceps* were obtained from GenBank (Accession Nos.: AF128500, AF128501). Novel sequences were deposited in GenBank (Accession Nos.: MG214260–MG214264). Uncorrected genetic distances were calculated using PAUP v. 4.0 (Swofford, 2002).

## RESULTS AND DISCUSSION

### Genetic distance and morphological characteristics of the southern Yunnan population

For the *ND*2 fragment analyzed, the two individuals from southeastern Yunnan Province are genetically identical to each other. The southern Yunnan individuals are 1.8% divergent from the topotypic *J. chapaensis*, 5.5% from the topotypic *J. yunnanensis*, 14.8% from *J. splendida*, and 18.2% from *J. flaviceps* (Table 1).

**Table 1:** Uncorrected genetic distances among members of sampled *Japalura sensu lato* using a fragment of 432 bp *ND*2 gene

	*Japalura splendida*	*Japalura flaviceps*	*Japalura yunnanensis*	*Japalura chapaensis* (topotype)	*Japalura chapaensis* (southern Yunnan)
*Japalura splendida*	–				
*Japalura flaviceps*	0.14977	–			
*Japalura yunnanensis*	0.18433	0.16359	–		
*Japalura chapaensis* (topotype)	0.17972	0.15668	0.0576	–	
*Japalura* chapaensis (southern Yunnan)	0.18203	0.14747	0.0553	0.01843	–

GenBank accession Nos. are listed in the methods.

Detailed morphometric and pholidosis characteristics are presented in Table 2. Morphological characteristics of the individuals from southern Yunnan resemble the diagnoses of *J. chapaensis* proposed by Ota (1992) (characteristics of the type and topotype from Ota [1992] are given in parentheses), SL 7 or 8 (7 or 8), IL 7–9 (7), MD 35–41 (35–37), and T4S 27–30 (28–30). Several morphological characteristics measured for the newly discovered population fall outside the currently recognized range of *J. chapaensis*, including relative length of Toe IV T4L/SVL 21.31%–22.35% (23.7%–26.0%), relative fore-limb length FLL/SVL 48.14%–50.79% (vs. 50.3%–54.7%), and relative hind limb length HLL/SVL 74.66%–77.81% (vs. 80.9%–81.4%). However, all characters closely match those of Ota (1992), with the same level of intraspecific phenotypic variation observed for other lineages in the genus *Japalura sensu lato* (Ota, 2000; Wang et al., 2017). With only two specimens known previously for *J. chapaensis*, it is expected that distinct populations of the species would possess some degree of variation among phenotypic characters.

**Table 2:** Morphological and pholidosis characteristics of *Japalura chapaensis*

Catalog No.	KIZ 034922	KIZ 034921	KIZ034923	MHNP 1948.45	KUZ 20097
Sex	M	F	F (subadult)	F	M
Type Status	–	–	–	Holotype	Topotype
SVL	67.10	68.50	50.80	59.60	58.10
TAL	168	157	109	122 (?)	/
HL	21.00	22.80	17.20	19.30	19.40
HW	13.80	14.40	11.30	/	/
HD	12.80	12.70	9.60	/	/
SEL	8.60	8.80	6.60	8.80	8.70
IOD	12.50	10.70	9.70	11.60	10.00
FLL	32.30	33.80	25.80	30.00	31.80
HLL	50.10	53.30	38.50	48.00	47.30
T4L	15.00	14.60	10.90	14.10	15.10
TRL	30.90	29.70	23.40	27.20	28.20
TAL/SVL	249.63%	228.61%	214.37%	204.70%	/
HL/SVL	31.30%	33.28%	33.86%	32.38%	33.39%
HW/HL	65.71%	63.16%	65.70%	/	/
SEL/HL	40.95%	38.60%	38.37%	45.60%	44.85%
FLL/SVL	48.14%	49.34%	50.79%	50.34%	54.73%
HLL/SVL	74.66%	77.81%	75.79%	80.54%	81.41%
TRL/SVL	46.05%	43.36%	46.06%	45.64%	48.54%
SL	8/8	7/7	8/7	7	8
IL	8/8	8/9	8/7	7	7
SOR	3/3	3/3	3/3	3	/
NSL	0	0	0	0	0
MD	35	41	36	35	37
F4S	23/23	25/26	23/24	22/23	24/22
T4S	27/28	28/29	30/30	27/28	30/30
PTS	2/2	2/1	2/2	/	/
PTY	3/3	3/2	3/2	/	/
PRS	6/7	9/7	10/8	/	/

Abbreviations are listed in the methods. Data of the holotype and topotype male were obtained from literature (Bourret, 1937; Ota 1989, 1992). “/” indicates missing data from the literature, and “–” indicates an incomplete tail. Tail of the holotype was recorded as complete, with its length noted in the original description; but it was shown to be incomplete in re-descriptions by Ota (1989, 1992).

Based on genetic and morphological data, we feel confident in the identity of the southern Yunnan population of *Japalura* as *J. chapaensis*; a new record for agamid lizards of China. To facilitate future study of this rare forest agamid and closely related species, we provide a detailed description of the newly collected individuals.

### Description of specimens from Yunnan (Figure 2)

**Figure 2:**
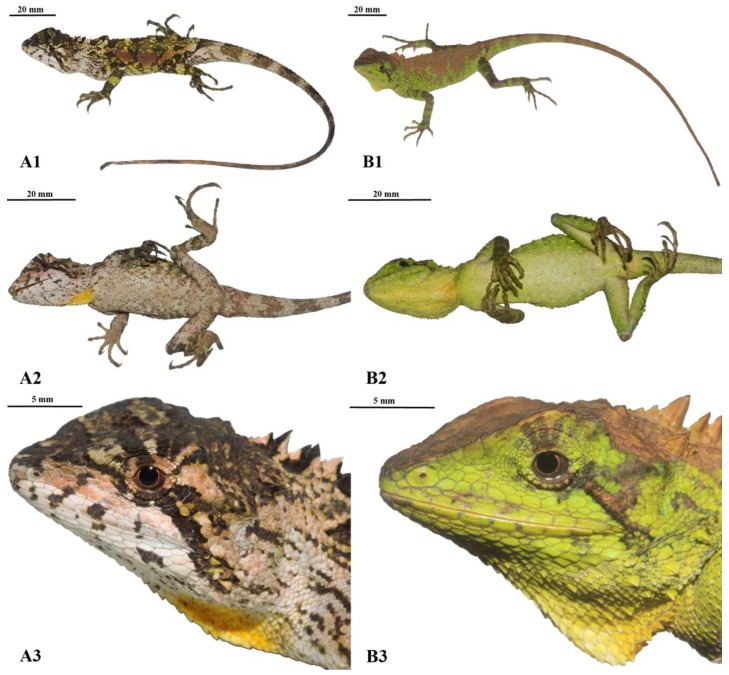
Photographs of (1) dorsal body view, (2) ventral body view, and (3) lateral head close-ups of male (A1–3; KIZ 034922) and female (B1–3; KIZ 034921) *Japalura chapaensis* in life (Photographs by Ke Jiang)

KIZ 034922, adult male; KIZ 034921, adult female; KIZ 034923, subadult female; all collected by Yufan Wang from Lvchun, southern Yunnan, China.

Description of southern Yunnan population.—Body size moderate, SVL 67.1 mm in adult male, 68.5 mm in adult female. Tail slender, long, TAL/SVL 249.63% in adult male, 228.61% in adult female, 214.37% in subadult female. Relative head length moderate, HL/SVL 31.30% in male, 33.28% in female, 33.86% in subadult female; HW/HL 65.71% in male, 63.16% in female, 65.70% in subadult female. Snout blunt, rounded; rostral rectangular, three times longer than height; nasal large, somewhat oval in shape, separated from rostral by single scale, in contact with supralabials; SL 7 or 8, elongated, weakly keeled; three suborbital scale rows from inferior orbit circle to sixth supralabial, middle row much enlarged; supraciliaries 6 or 7, imbricate, overlapping one-fifth to one-third of posterior one; numerous large, keeled scales forming single ridge from postorbital to supra-anterior tympanum on each side; tympanum concealed, covered by fine scales; two or three large conical scales posterior to tympanum. Mental pentagonal. IL 7–9, smooth; numerous large, conical postrictal scales present. Dorsal head scales heterogeneous in size and shape, strongly keeled; three large, hexagonal, conical scales arranged in triangular position on dorsal snout; interparietal scale elongate, hexagonal, parietal eye present; two pairs of distinct, conical scales present on raised areas posterior to interparietal on each side, forming w-shape ridge anterior to beginning of nuchal crest; single, large, conical, postorbital scale posterior to last supraciliaries; keeled, sub-pyramidal scales on occipital region 2 or 3, with one distinctively tall, enlarged. Ventral head scales keeled, more greatly keeled posteriorly, heterogeneous in size except for a few irregularly scattered large scales, particularly on lateral surfaces; gular pouch present, distinct in life; longitudinal gular fold present in life; transverse gular fold absent.

Dorsal body scales strongly keeled, heterogeneous in size and shape; scales of axillary region smaller, circular shaped; shoulder fold present, short, weakly defined; enlarged scales scattered on dorsal body, moderately raised, each bearing a single distinct keel; some enlarged scales arranged in paravertebral row close to vertebral crest; mid-dorsal crest scales 35–41, larger than neighboring scales; nuchal crest scales 5–8, greatly enlarged, triangular shaped; dorsal crests relatively lower, serrated. Ventral body scales keeled, largely homogeneous in size; enlarged scales scattered ventrolaterally. Limb scales keeled, largely homogeneous in size on fore-limbs, more heterogeneous in size on hind limbs; enlarged, keeled scales scattered irregularly on posterior hind surfaces and crus. Tail slender, scales strongly keeled.

### Coloration in life (Figure 2)

Color codes follow Köhler (2012). Both males and females of *J. chapaensis* are sexually dichromatic. For males, the background color of head varies from chamois (code 84) to olive sulphur yellow (code 90). Four dark brownish olive (code 127) to dusky brown (code 285) transverse bands span the dorsal surface of the head, two of which are located on the snout, one between the eyes and one posterior to the eyes. The background coloration of the lateral surfaces of the head are light buff (code 2) to light flesh color (code 250). Nine dusky brown (code 285) stripes radiate out around each eye. The two posterior-directing radial stripes are the broadest, with the superior one extending from the posterior corner of the eye to the anterior tympanic region, and the inferior one extending to the corner of the mouth. The subocular radial stripes do not extend outside of the circular orbit. Three to four dusky brown (code 285) blotches are scattered on both the supralabials and infralabials. The oral cavity and tongue are dark spectrum yellow (code 79).

The background color of the neck and axillary region is light flesh color (code 250), which transitions to olive sulphur yellow (code 90), yellow green (code 103), and eventually citrine (code 119) posteriorly. Most regions of the dorsal and lateral surfaces of the body possess dusky brown (code 285) reticulated patterns around the axillary region and blotches along the dorsal midline. An irregularly shaped dorsolateral stripe is present on each side of the body, consisting of three Pratt’s rufous (code 72) colored, elongated blotches, running posterolaterally away from the vertebral midline. The coloration of these stripes can change to chamois (code 84) depending on the condition or mood of the lizards. Five small, sulphur yellow (code 80) blotches are evenly scattered along the dorsal midline from the shoulder to the pelvis. The dorsal surfaces of the limbs are olive sulphur yellow (code 90), with numerous dusky brown (code 285) bands spanning the upper surfaces of the limbs to the digits. A pale buff (code 1) stripe is present on the posterior surfaces of the upper hind limbs. The tail is pale buff (code 1), with six citrine (code 119) bands spanning about one-third of its total length. The bands of the tail extend to the ventral surface, forming complete rings around the tail. The remaining posterior surfaces of the tail are uniform ground cinnamon (code 270) in color.

The ventral surface of the head is pale buff (code 1), with some dusky brown (code 285) speckles scattered randomly. A distinct dark spectrum yellow (code 78) gular spot is present in the center of the gular region. The background color of the ventral surface of the body and limbs is pale horn (code 11) anteriorly, with the coloration changing to light pale pinkish buff (code 3) posteriorly, including the upper ventral surfaces of the limbs and the ventral surfaces of the tail. Grayish horn (code 268) speckles are scattered across the ventral surfaces of the body and limbs.

Female body coloration differs from male coloration (described above) as follows. The dorsal surfaces of the head and neck are mostly uniform clay (code 18) in coloration. The lateral surfaces of the head are yellow-green (code 103), with two to four clay (code 18) colored radial stripes extending from around the eyes. Five rectangular clay (code 103) blotches are scattered evenly along the dorsal surface of the body at the midbody, with all blotches loosely connected along the dorsal midline. The background color of the lateral body surfaces, dorsal limb surfaces, and dorsal tail surface are pistachio (code 102). A pale buff (code 1) stripe is present posteriorly on each upper hind limb. Clay (code 18) bands are present on the first one-third of the tail; however, the bands extend to the lateral surfaces of the tail only, and do not form complete rings. The posterior two-thirds of the tail is uniform clay (code 18).

The ventral surface of the head is uniform light pistachio (code 101) with no speckles. This coloration transitions gradually to dark spectrum yellow (code 79) toward the center of the gular region, forming a gular spot. The ventral surfaces of the body, limbs, and tail are pale green (code 99).

### Ecology and distribution

**Figure 3:**
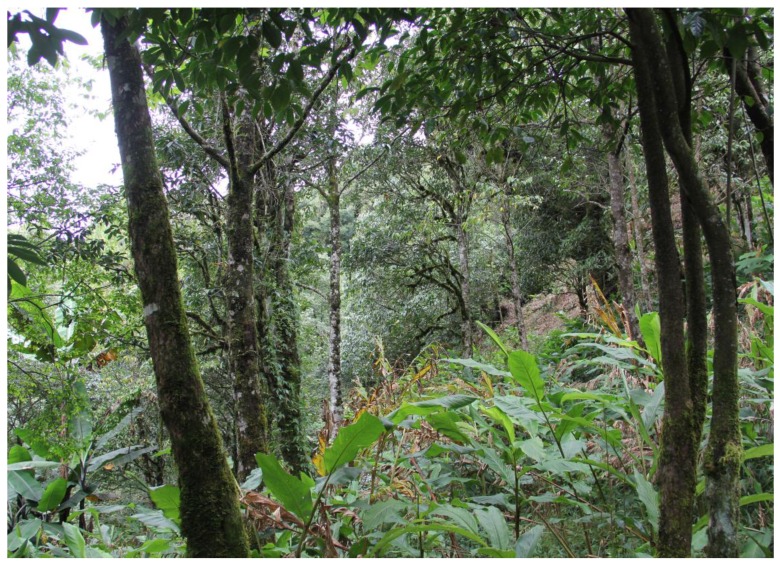
Habitat of *Japalura chapaensis* in Lvchun, southeastern Yunnan Province, China (Photograph by Yu-Fan Wang)

*Japalura chapaensis* is arboreal, inhabiting low shrubs in tropical forest, particularly near streams and along forest edges (Figure 3). In Lvchun, individuals are usually found resting on fern leaves at night. Currently, the species is known from Lvchun County of Honghe Prefecture, Yunnan Province, China, and from Cao Bang, Hai Duong, and Lao Cai Provinces of Vietnam (Ananjeva et al., 2007). In addition, morphologically similar individuals have been photographed in Daweishan Natural Reserve of Honghe Prefecture, 130 km east from Lvchun County, which suggests that the species has a wider geographic distribution along the China-Vietnam border (Jian Wang and Shuo Qi, personal communications).

### Diagnoses from congeners

Previous studies have compared *J. chapaensis* with five island-congeners (*J. brevipes*, *J. makii*, *J. mitsukurii* [synonym of *J. swinhonis*], *J. polygonata xanthostoma*, and *J. swinhonis*), and two mainland-congeners (*J. splendida* and *J. yunnanensis*) (Ota, 1989; Ota & Weidenhöfer, 1992). Based on their results, previous authors concluded that *J. chapaensis* is morphologically most similar to *J. brevipes*, *J mitsukurii*, *J. swinhonis*, and *J. yunnanensis*, but can be distinguished from these lineages by its relative head length, relative snout-eye length, Toe IV length, and Toe IV subdigital lamellae and middorsal crest scale counts (Ota, 1989; Ota & Weidenhöfer, 1992).

With the addition of the new collections from southern Yunnan and a more comprehensive examination of congeners, we found that *J. chapaensis* can be readily differentiated from all five Taiwanese species by having much larger nuchal crest scales and a distinct head shape with a w-shaped lump on the occipital region anterior to the nuchal crest. In addition, we found *J. chapaensis* to be morphologically most similar to *J. yunnanensis*, with both species lacking a transverse gular fold, and possessing concealed tympanum, large nuchal crests, irregularly distributed and enlarged ventrolateral scales, dark spectrum yellow gular spots in males (sometimes in females), and jagged dorsolateral stripes that are oriented in a posterolateral direction away from the dorsal midline. However, although phenotypically similar, *Japalura chapaensis* differs from *J. yunnanensis* by having a tendency toward fewer mid-dorsal crest scales (MD 35–41 vs. 39–46), and relatively shorter tails (TAL/SVL 249.63% in male, 204.70%–228.61% in females vs. 255.19%–288.81% in males, 237.45%–259.67% in females) (Table 2).

For the remaining mainland congeners from non-Himalayan regions, *J. chapaensis* differs from *J. fasciata* by having a longer tail (TAL >204% SVL vs. <180%) and longer hind limbs (HLL >74% SVL vs. <69%), and by the absence of a transverse hourglass-shaped marking on the dorsum (vs. present); from *J. batangensis*, *J. brevicauda*, *J. dymondi*, *J. flaviceps*, *J. grahami*, *J. iadina*, *J. laeviventris*, *J. splendida*, *J. vela*, *J. yulongensis*, and *J. zhaoermii* by having significantly enlarged nuchal crest scales (vs. not significantly differentiated from dorsal crest scales), bright yellow oral cavity (vs. flash color or blackish blue), and by the absence of a transverse gular fold (vs. present); from *J. varcoae* by having a concealed tympanum (vs. exposed) and enlarged, differentiated nuchal crest scales (vs. not significantly differentiated); and from *J. micangshanensis* by having significantly enlarged nuchal crest scales (vs. not significantly differentiated from dorsal crest scales) and by the presence of gular spots (vs. absent).

In conclusion, *Japalura chapaensis* can be diagnosed from its congeners on the basis of the following set of characters: (1) body size moderate SVL 58.10–68.50 mm; (2) tail relatively long TAL/SVL 249.63% (male), 204.70%–228.61% (females); (3) limb length moderate, FLL/SVL 48.14%–54.73%, HLL/SVL 74.66%–81.41%; (4) short, w-shaped ridge present on occipital head anterior to nuchal crest; (5) tympanum concealed; (6) supraciliaries overlapping less than one-third of posterior one; (7) NSL absent (8) lateral gular fold and gular pouch present, well developed; (9) transverse gular fold absent; (10) nuchal crest scales large, crest height/HL 10.48%–14.04%; (11) MD 35–41; (12) T4S 28–30; (13) ventral scales heterogeneous in size with enlarged scales scattered ventrolaterally; (14) ground body coloration camouflaged with yellow-green, clay, and dusky brown coloration in life; (15) dorsolateral stripes present in males, jagged, sometimes discontinuous, posterolaterally away from the dorsal midline; (16) gular spot present, distinct in males, somewhat faded in females, dark spectrum yellow in both sexes; and (17) oral cavity and tongue dark spectrum yellow in life.

Despite our discovery of a new population of *J. chapaensis* in southern China, the small number of individuals seen in the wild continues to limit our understanding of this rare *Japalura* species in northern Indochina. Future surveys and greater international collaborations throughout this region are needed to clarify the morphological variation, distribution patterns, and population-level genetic diversity of *J. chapaensis*.

## APPENDIX

### Specimens examined

***Japalura batangensis*** (*n*=16): CIB 1902–1908, 2227, 2233, 2243, KIZ 84011, 801081, Batang, Sichuan, China; KIZ 09404, 019311, 019312, KIZ 019314, Mangkang, Tibet, China.

***Japalura dymondi*** (*n*=7): CIB 1869, 87234, Panzhihua, Sichuan, China; KIZ 95I1001, 1002, 1016, 1018, 1022, Dayao, Yunnan, China.

***Japalura fasciata*** (*n*=3): CIB 2620, 2621, 2622, Pen Hsien, Sichuan Province, China.

***Japalura flaviceps*** (*n*=13): CIB 2234, 2332, 2333, 2341, 2354, 2355, 2549, 2554, 2556, 2561, 2567; KIZ 05181, 05182; Luding, Sichuan, China.

***Japalura grahami*** (*n*=1): USMN 65500 (holotype), Yibin, Sichuan, China.

***Japalura makii*** (*n*=2): MCZ R-172743 (paratype), R-181443, Taiwan, China.

***Japalura micangshanensis*** (*n*=9): CIB 86348, 86351, Xianyang, Shaanxi, China; CIB 86356, 86357, 86360, 86361, Luonan, Shaanxi, China; CIB 2572, 2578, 2582, Wenxian, Gansu, China.

***Japalura polygonata*** (*n*=3): CAS 21215, 21243, 21244, Kagoshima Prefecture, Japan.

***Japalura splendida*** (*n*=6): USNM 35522 (holotype), Yichang, Hubei, China; CIB 2588, 2591, 2596, 72468, 72469, Chongqing, China.

***Japalura swinhonis*** (*n*=4): CAS 18085, 18089, 18098, 18099, Taiwan, China.

***Japalura varcoae*** (*n*=3): CIB 2650, 2651, KIZ 85II0006, Kunming, Yunnan, China.

***Japalura vela*** (*n*=11): KIZ 013801 (holotype), KIZ 013802, 013813, 013800, 013805–013811 (paratopotypes), Jerkalo, Tibet, China.

***Japalura yunnanensis*** (*n*=8): CIB 2684, 2686, 2687, 2689, KIZ 82081, Longling, Yunnan, China; KIZ 74II0240, 0248, 79I469, Tengchong, Yunnan, China.

***Japalura zhaoermii*** (*n*=14): CIB 2690 (holotype), 86432, 86435, 85721, 85722, 86433, 86434, 86436, Wenchuan, Sichuan, China; CIB 2232, 2244, 2240, KIZ 84032, 85030, Lixian, Sichuan, China.
